# Human S100A9 Protein Is Stabilized by Inflammatory Stimuli via the Formation of Proteolytically-Resistant Homodimers

**DOI:** 10.1371/journal.pone.0061832

**Published:** 2013-04-23

**Authors:** Matteo Riva, Zhifei He, Eva Källberg, Fredrik Ivars, Tomas Leanderson

**Affiliations:** 1 Immunology Group, Lund University, Lund, Sweden; 2 Active Biotech AB, Lund, Sweden; Carl-Gustav Carus Technical University-Dresden, Germany

## Abstract

S100A8 and S100A9 are Ca^2+^-binding proteins that are associated with acute and chronic inflammation and cancer. They form predominantly heterodimers even if there are data supporting homodimer formation. We investigated the stability of the heterodimer in myeloid and S100A8/S100A9 over-expressing COS cells. In both cases, S100A8 and S100A9 proteins were not completely degraded even 48 hrs after blocking protein synthesis. In contrast, in single transfected cells, S100A8 protein was completely degraded after 24 h, while S100A9 was completely unstable. However, S100A9 protein expression was rescued upon S100A8 co-expression or inhibition of proteasomal activity. Furthermore, S100A9, but not S100A8, could be stabilized by LPS, IL-1β and TNFα treatment. Interestingly, stimulation of S100A9-transfected COS cells with proteasomal inhibitor or IL-1β lead to the formation of protease resistant S100A9 homodimers. In summary, our data indicated that S100A9 protein is extremely unstable but can be rescued upon co-expression with S100A8 protein or inflammatory stimuli, via proteolytically resistant homodimer formation. The formation of S100A9 homodimers by this mechanism may constitute an amplification step during an inflammatory reaction.

## Introduction

S100A8 and S100A9 proteins belong to the S100 protein family, which constitutes over 20 low molecular Ca^2+^-binding proteins [1], [2]. In particular, S100A8 and S100A9 are the focus of intense research, as they are associated with several inflammatory diseases and cancer [3], [4]. In addition, they are well known protein markers for an increasing number of inflammatory disorders such as rheumatoid arthritis, inflammatory bowel disease and prostate cancer [5]–[7].

S100A8 (10 kDa) and S100A9 (14 kDa) proteins are expressed constitutively in circulating neutrophils and monocytes and their expression can be induced in resting tissue macrophages [8]–[12]. Although it has been documented that S100A8 and S100A9 can exist both as homo- and heterocomplexes, they preferentially form heterodimers or heterotetramers in a Ca^2+^ and Zn^2+^ dependent way [13]–[18]. Recent studies suggested that S100A8/S100A9 homo- and hetero-oligomers may have distinct roles in cell physiology. In particular, it has been reported that S100A8/S100A9 heterocomplexes could mediate apoptosis [19], induce neutrophil chemotaxis [20], activate the NFêB pathway [21], exhibit antimicrobial activity [22] and regulate NADPH oxidase activation upon arachidonic acid binding [23].

S100A8 knock-out (KO) mice show a lethal phenotype [24], while S100A9-KO mice are perfectly viable and no major differences in inflammatory response have been observed compared to wild type animals [25]. S100A9, but not S100A8 could bind heparansulphate [26], mediate neutrophil adesion to fibronectin [27] and increase Mac-1 affinity [28]. Furthermore, it has been shown that S100A9 protein is involved in tumour growth [29].

In the present work, we investigated the protein turnover and stability of S100A8/S100A9 homo- and heterodimers. In particular, we observed that human S100A9 (hS100A9) homodimers were unstable and readily degraded, while human S100A8 (hS100A8) homodimers were perfectly stable. Interestingly, S100A9 expression could be rescued upon challenge with inflammatory stimuli that stabilized the protein. Those findings showed that the cells could generate a qualitatively different S100A8/S100A9 oligomeric form in response to proper stimuli.

## Materials and Methods

### Cell Culture

The human monocytic leukemia cell line THP-1 was grown in RPMI 1640 culture medium (Invitrogen, UK) supplemented with 10% fetal bovine serum (FBS; Invitrogen, UK), 2 mM Glutamine (Sigma-Aldrich, USA), 1 mM sodium pyruvate, 10 mM Hepes, 100 U/ml of penicillin and 100 µg/ml of streptomycin (P/S; Invitrogen, UK), at 37°C in 5% CO_2_. COS and LEP cells were cultured in Dulbecco’s Modified Eagle Medium (DMEM) supplemented with 10% fetal bovine serum (Invitrogen, UK), 2 mM Glutamine (Sigma-Aldrich, USA), 100 U/ml of penicillin and 100 µg/ml of streptomycin (Invitrogen, UK) at 37°C in 5% CO_2_. LEP cells were cultured in the presence of Non-Essential-Aminoacids (NEA; Sigma-Aldrich, USA). LEP cell is an embryonic fibroblast cell line kindly provided by Prof. Vera Casslen [30].

### COS and LEP Cell Transfection and Treatment

Three different transfections were performed:

1. hS100A8∶1 µg pcDNA3.1-hS100A8+1 µg pcDNA3.1-EGFP;
2. hS100A9∶1 µg pDream2.1-hS100A9+1 µg pcDNA3.1-EGFP;
3. hS100A8/hS100A9∶1 µg pcDNA3.1-hS100A8+1 µg pDream2.1-hS100A9.

Briefly, COS or LEP cells were seeded in 24 well plate in serum-free medium the day before transfection. DNA-Lipofectamine 2000 mixture was prepared as follow: 2 µl Lipofectamine 2000/reaction were incubated in 50 µl Optimem medium for 5 min at room temperature (RT). Plasmid DNA was added to the mixture and incubated for further 20 minutes at RT. Subsequently, the DNA-Lipofectamine 2000 mixture was added to the cells, which were incubated for 3 hrs at 37°C. Finally, 1 ml/well of medium was added to the transfected COS cells and a further 24 h incubation at 37°C was performed.

After overnight incubation, transfected COS cells were treated with different concentrations of proteasomal inhibitor MG132 (Millipore, Billerica, MA, USA), IL-1β (Invivogen, San Diego USA), LPS (Invivogen, San Diego, USA) and TNFα (Invivogen, San Diego, USA) as indicated in Figure Legends. In some experiments, to study protein dimerization, transfected COS cells were washed in PBS and incubated for 30 min on ice with 1 mM disuccinimidyl suberate (DSS, Sigma-Aldrich, USA) dissolved in PBS. To study protein stability, transfected COS cells were treated with 100 µg/ml of cycloheximide.

### Western Blot

10 µg of total cell extract was loaded into 4–20% polyacrylamide gel (BioRad, Solna, Sweden). Proteins were subsequently transferred to PVDF membrane (Roche, Mannheim, Germany), which was saturated with 1% dry milk in PBS-Tween 0.05%. Membranes were incubated with the appropriate primary antibody diluted 1∶5000 in PBS-Tween overnight at 4°C, washed 3 times in PBS-Tween, incubated for 1 h at RT with Goat anti-Rabbit or anti-Mouse secondary antibodies (Abcam, Cambridge, UK) diluted 1∶5000, then washed 3 times in PBS-Tween and finally developed using ECL kit from Roche, (Mannheim, Germany).

The primary antibodies were the following: Mouse anti-human S100A9 (Novus Biologicals Inc., CO, USA), Rabbit anti-human S100A8 (kindly provided by Prof. Nancy Hogg, UK) and Rabbit anti-human β-tubulin (Novus Biologicals Inc., CO, USA).

### Immunoprecipitation (IP)

Five million COS cells were co-transfected with pcDNA3.1-hS100A8 and pDream2.1-hS100A9, as described above. After washing, the cells were re-suspended in lysis buffer (75 mM Tris/HCl pH 7.6, 1.25% NP-40, 100 mM NaCl and complete protease inhibitors) and cell debris removed by centrifugation. The supernatants were thereafter pre-cleared upon incubation with streptavidin (hS100A9) or protein G-conjugated beads (hS100A8) at 4°C for 1 hour with gentle shaking. Subsequently, the supernatants were incubated with biotinylated mouse anti-hS100A9 antibody (43/8-bio, produced in our lab) or rabbit anti-hS100A8 antibody (kind gift of Prof. Nancy Hogg, UK), at 4°C overnight. The following day, streptavidin- or protein G-conjugated beads (Invitrogen, UK) were added to the samples at 4°C for 1 hour. Subsequently, beads-protein complexes were harvested, washed 3 times in washing buffer (Invitrogen, UK), eluted in Laemmli Sample Buffer (BioRad, Solna, Sweden) and boiled at 70°C for 10 min. The samples were then analyzed by SDS-PAGE and Western blot. In some experiment, as a control, COS cells were transfected only with pDream2.1-S100A9, followed by the same procedure described above. TRIS buffer, NaCl and NP-40 were purchased from Sigma-Aldrich, (USA) while complete protease inhibitors were obtained from Roche (Mannheim, Germany).

## Results

### The hS100A8/hS100A9 Heterodimer is a More Stable form as Compared to hS100A8 or hS100A9 Expressed Alone

**Figure 1:**
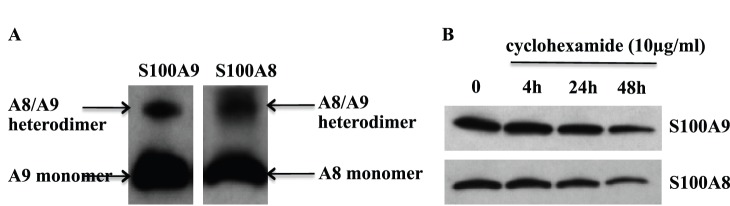
hS100A8 and hS100A9 form a stable heterodimer in THP-1. THP-1 were treated with DSS for 30 min in ice. Subsequently samples were used for Western blot and stained for hS100A9 and hS100A8 (**A**). THP-1 cells were treated with 10 µg/ml cycloheximide for 4 h, 24 h and 48 h. Samples were, then, collected and Western blot for hS100A9 and hS100A8 was performed (**B**).

In order to investigate which S100A8/S100A9 oligomers were expressed in monocytes, we treated THP-1 cells with the cell-permeable cross-linker DSS. By Western blot, we could notice a band around 24 kDa, which was representative of the S100A8/S100A9 heterodimer complex, while we could not detect any homodimeric forms (Fig. 1a). Previously, it has been shown that in granulocytes the S100A8/S100A9 heterocomplex was extremely protease resistant [31]. We confirmed this finding by treating THP-1 cells, for different periods of time, with the protein translation inhibitor cycloheximide and, indeed, found that the S100A8/S100A9 heterodimer was not completely degraded even after 24 h (Fig. 1b).

**Figure 2:**
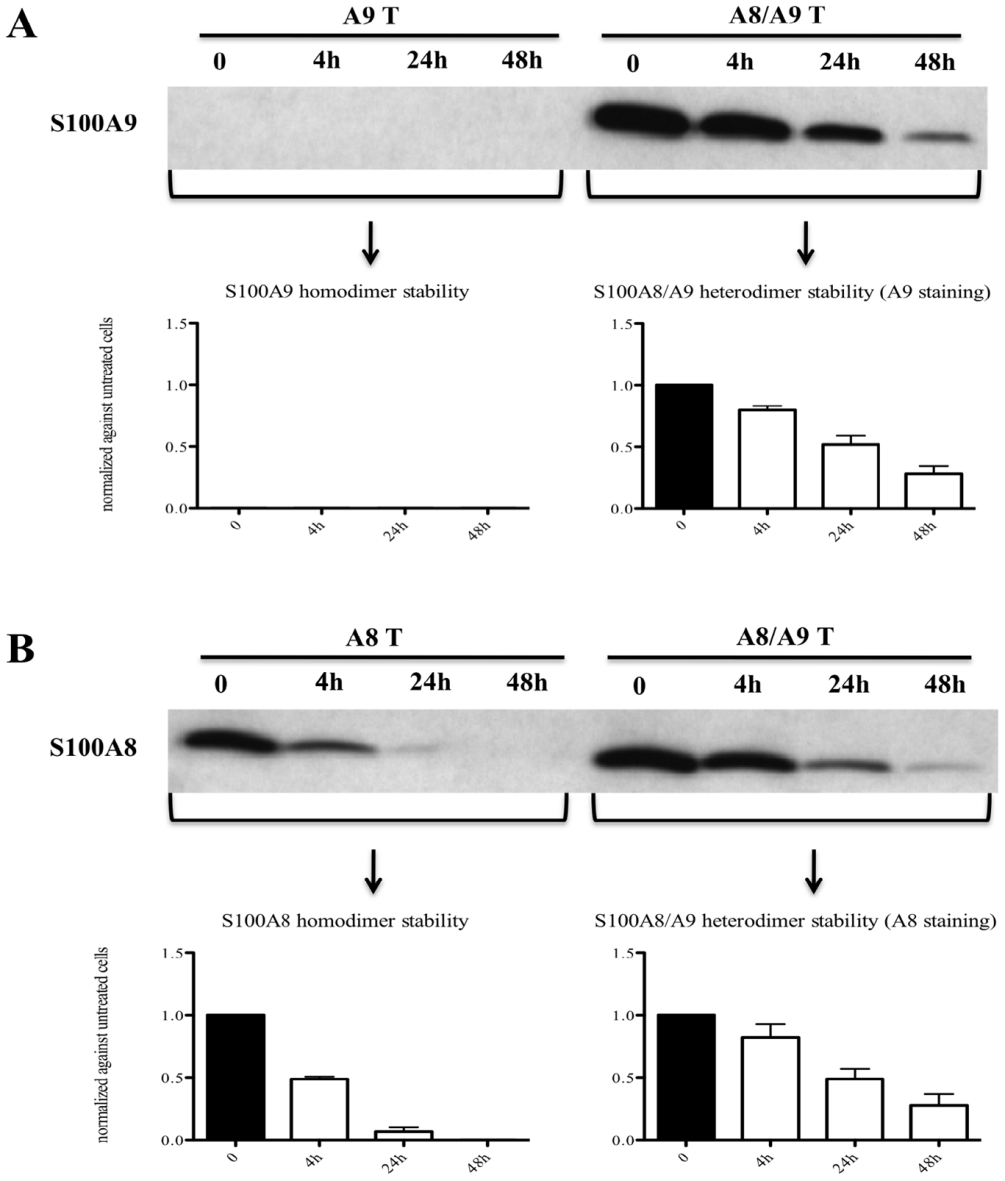
The hS100A8/hS100A9 heterodimers were more stable than hS100A8 and hS100A9 homodimers. COS cells were transfected with hS100A8 and hS100A9 separately or together. After 24 h, COS cells were treated with 100 µg/ml cycloheximide for 4 h, 24 h and 48 h. Samples were collected and analyzed by Western blot. Filters were stained with (**A**) anti-hS100A9 or (**B**) anti-hS100A8. In the lower charts, the relative band intensity compared to non-stimulated cells are indicated.

Since we were able to detect only S100A8/S100A9 heterodimers in THP-1 cells, we decided to overexpress hS100A8 and hS100A9 in COS cells in order to investigate the stability of S100A8 and S100A9 individually. The results in Fig. 2a–b indicated that the hS100A8/hS100A9 heterodimer is the most stable form since hS100A8 expressed alone decayed within 24 hrs. Surprisingly, hS100A9 expressed alone was not detectable even at the earliest time point, suggesting that hS100A9 was unstable and immediately degraded.

In both experiments, we checked cell viability and even though both THP-1 and COS cells stopped proliferating upon cycloheximide treatment, they did not die (Fig. S1a–b).

### The hS100A9 Protein is Proteolytically Degraded in COS Cells

**Figure 3:**
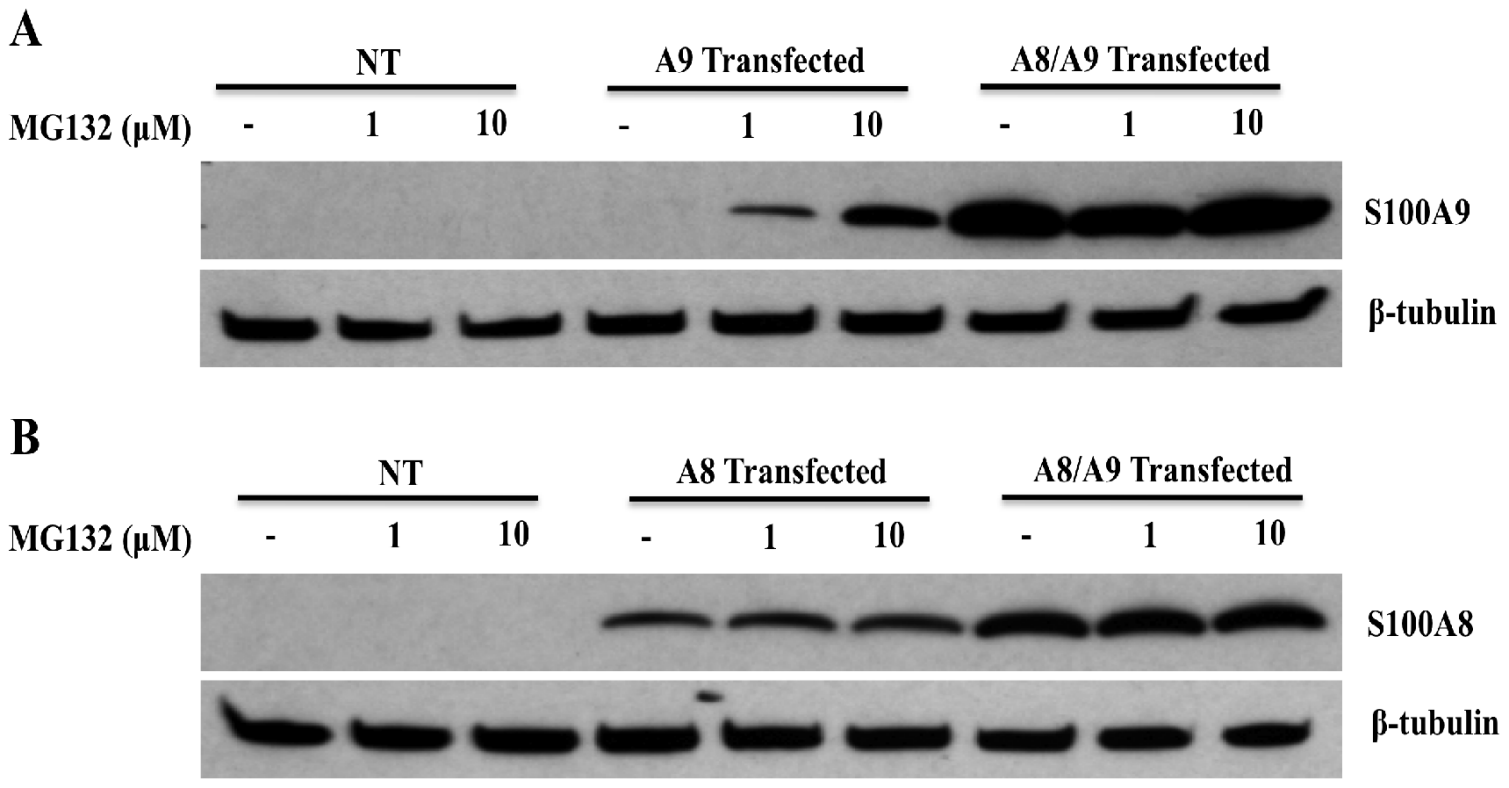
hS100A9 protein was unstable in COS cells but could be stabilized by MG132 or co-transfection with hS100A8. COS cells were transfected with hS100A8 and hS100A9 constructs either separately or together. 24 h after transfection, the cells were stimulated for 8 h with 1 or 10 µM MG132 and subsequently analyzed by Western blot using (**A**) anti-hS100A9 or (**B**) anti-hS100A8. The first three lanes (**NT**) of each panel represented non-transfected cells. From lane 4 to 6, COS cells were transfected with (**A; A9 Transfected**) hS100A9 or (**B; A8 Transfected**) hS100A8 separately, while from lane 7 to 9 (**A8/A9 Transfected**) cells were co-transfected.

To corroborate our hypothesis about the rapid turnover of hS100A9 protein, we treated the hS100A9-transfected cells with increasing concentrations of the proteasomal inhibitor MG132 and observed a marked increase of hS100A9 protein expression (Fig. 3a). Thus, hS100A9 expressed alone is unstable and subjected to rapid proteolytic degradation. In contrast, when COS cells were co-transfected with both hS100A8 and hS100A9 constructs, we observed a robust increase in hS100A9 protein expression (Fig. 3a), indicating that hS100A8 protein could rescue the hS100A9 protein from proteasomal degradation.

On the other hand, hS100A8 was readily detectable in the cells transfected with the hS100A8 construct alone. In this case, expression was not altered by MG132 addition, indicating that the hS100A8 protein was stable by itself (Fig. 3b). In addition, also the level of hS100A8 protein was increased, even though to a minor extent, when co-expressed with hS100A9 (Fig. 3b).

### The hS100A9 Protein is Unstable Also in Fibroblasts

**Figure 4:**
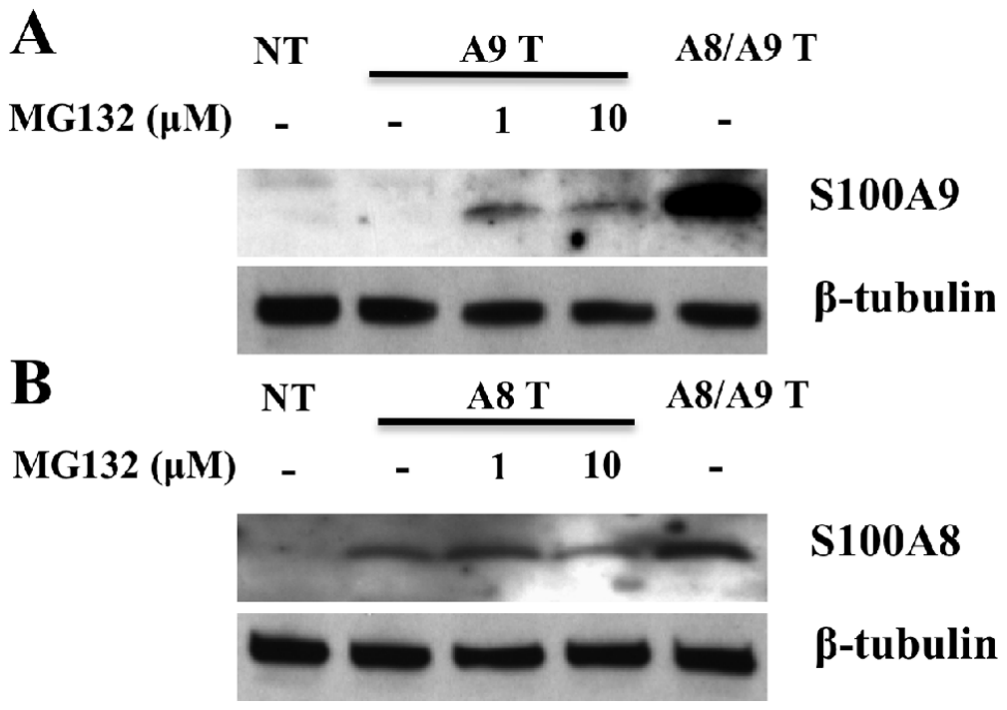
hS100A9 protein was unstable in LEP cells but could be rescued by MG132 and hS100A8. hS100A8 and hS100A9 expression vectors were transfected into human fibroblasts (LEP cells) as described before for COS cells. After SDS-PAGE and Western blots were performed either with (**A**) anti-hS100A9 or (**B**) anti-hS100A8. Lane 1 (**NT**) represented non-transfected cells; from lane 2 to lane 4 cells were transfected with hS100A9 (**panel** **A;** **A9 T**) or with hS100A8 (**panel B**; **A8 T**); in lane 5 (**A8/A9 T**) cells were co-transfected with both constructs.

Next we wanted to determine whether the instability of hS100A9 protein was peculiar to the COS cells. For this purpose, we transfected a human lung embryonic fibroblast cell line (LEP) (Fig. 4a–b) with hS100A8 and hS100A9 constructs, alone or together. The results showed that hS100A9 was proteolytically degraded also in this cell line. The protein could again be rescued upon MG132 treatment or hS100A8 protein co-expression. Also in these cells, hS100A8 was stable by itself. Taken together, these data indicated that the instability of hS100A9 protein is not tissue-specific.

### Formation of hS100A8/hS100A9 Heterodimers Stabilizes the hS100A9 Protein

**Figure 5:**
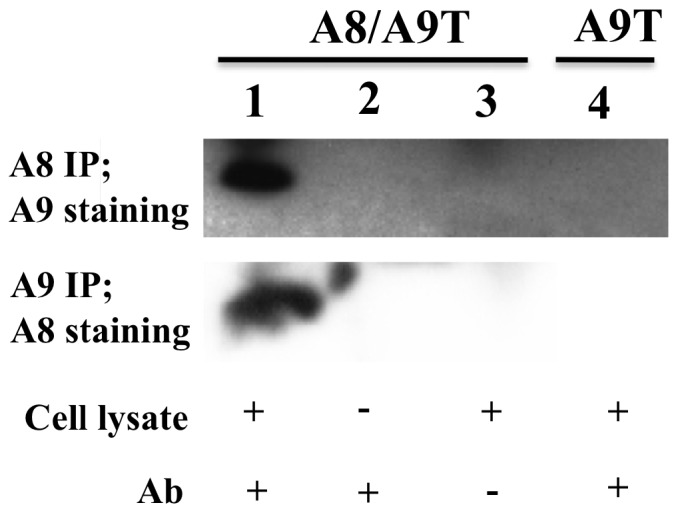
hS100A8 associates with hS100A9 in co-transfected COS cells. COS cells were co-transfected with expression vectors for both hS100A8 and hS100A9. 24 h later, hS100A9 was immunoprecipitated and analyzed for hS100A8 expression by Western blot (Panel 2, lane 1). The same experiment was repeated immunoprecipitating hS100A8 and staining for hS100A9 (Panel 1, lane 1). Lane 2 and 3 represented the controls. In brief, in control samples, COS cells were co-transfected and a full Co-IP experiment was performed but without the cell extract, or the antibody, respectively. In panel 1, lane 4, COS cells were transfected only with hS100A9-carrying vector. The hS100A9 protein was immunoprecipitated and Western blot performed with hS100A8 staining.

From the data shown above, we hypothesized that the stabilization of hS100A9, observed upon co-expression with S100A8, might be due to formation of S100A8/A9 heterodimers. To confirm this, we co-transfected COS cells with both the hS100A8 and hS100A9 constructs and performed a co-immunoprecipitation (Co-IP) experiment, followed by Western blotting. We observed that, upon Co-IP of hS100A9, we could effectively detect the hS100A8 partner and vice versa (Fig. 5). Thus, hS100A9 was rescued from proteasomal degradation by forming complexes with hS100A8.

### Inflammatory Stimuli Stabilize the hS100A9 Protein

**Figure 6:**
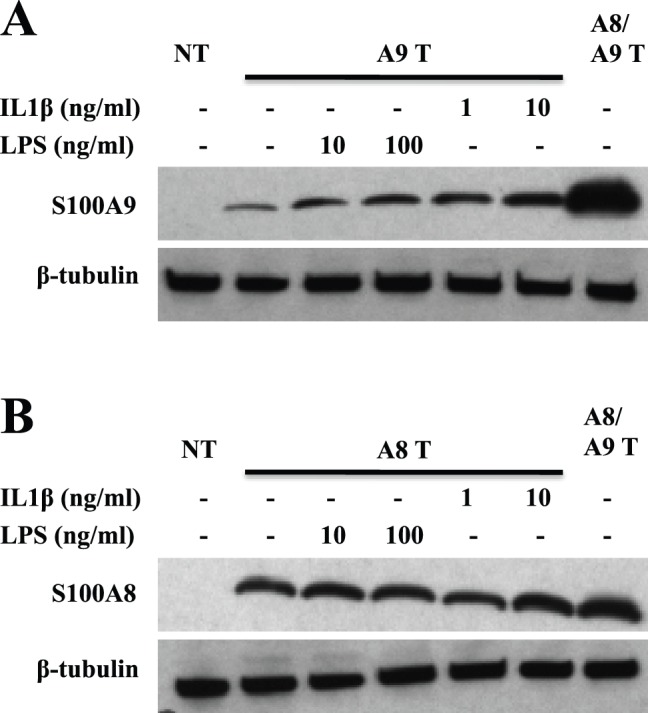
LPS and IL1β induced hS100A9 protein stabilization. COS cells were transfected either with hS100A8 or hS100A9 constructs, as described above. 24 h after transfection, COS cells were stimulated with either 1 or 10 ng/ml IL1β or alternatively with either 10 or 100 ng/ml LPS. Samples were collected and analyzed by Western blot. Filters were stained with (**A**) anti-hS100A9 and (**B**) anti-hS100A8. Lane 1 (**NT**) represented non-transfected cells; from lane 2 to 6, COS cells were transfected with (**A**; **A9 T**) hS100A9 or (**B**; **A8 T**) hS100A8 separately; in lane 7 (**A8/A9 T**) COS cells were co-transfected with both plasmids.

Expression of S100A9 protein could, under inflammatory conditions, be detected in certain tissue cells such as keratinocytes or chondrocytes [32], [33]. Therefore, we wanted to test whether pro-inflammatory stimuli could promote hS100A9 stability. For this purpose we treated hS100A9-transfected COS cells with IL-1β or LPS. As shown in Fig. 6a–b, both LPS and IL-1β were able to markedly increase hS100A9 but not hS100A8 expression. In addition, we confirmed our finding treating COS cells with TNFα, showing that hS100A9 stabilization was not peculiar to IL1β but it could occur also upon challenge with other pro-inflammatory stimuli (Fig. S2). The fact that also inflammatory stimuli promote hS100A9 expression, suggest that hS100A9 could escape proteasomal degradation in response to external signals.

### IL1β Promotes the Formation of Protease-resistant hS100A9 Homodimers

**Figure 7:**
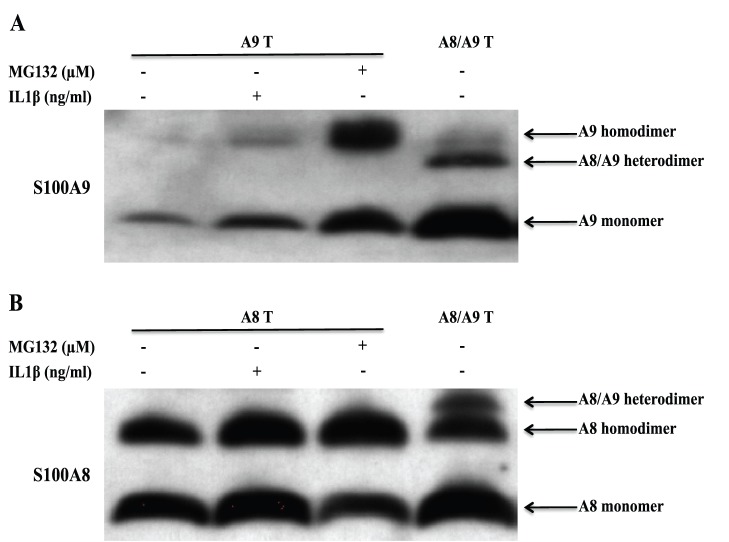
IL1β promotes the formation of protease-resistant hS100A9 homodimers. COS cells were transfected with hS100A8 and hS100A9 expression vectors, either separately or together. 24 h after transfection, transfected cells were treated either with MG132 or IL1β. Then, COS cells were incubated with 1 mM DSS on ice for 30 minutes and analyzed by Western blot (hS100A9 (**A**) and hS100A8 (**B**)).

To further analyze the composition of the different S100A8/S100A9 complexes, we transfected COS cells with hS100A8 and hS100A9 cDNA constructs alone or together. We thereafter treated COS cells with the membrane permeable cross-linker DSS and analyzed the S100 proteins by Western blotting staining for hS100A9 (Fig. 7a) or hS100A8 (Fig. 7b). When hS100A8 was expressed alone (Fig. 7b), we observed a band with an approximate Mw of 20 kDa representing the hS100A8 homodimer. When hS100A8 was expressed together with hS100A9, we observed an additional band at 24 kDa, representing the S100A8/S100A9 heterodimer. The 24 kDa heterodimer band was weaker than the 20 kDa hS100A8 homodimer band.

When hS100A9 was expressed alone and the cells were treated with MG132 to prevent its degradation (Fig. 7 a), we observed a band with an approximate Mw of 28 kDa, representing the hS100A9 homodimer. When hS100A8 and hS100A9 were co-transfected, we could only notice a S100A8/S100A9 heterodimer band at 24 kDa.

Lastly, we wanted to investigate if this event was peculiar for MG132 or if also inflammatory stimuli, such as IL1β, could promote protease-resistant hS100A9 homodimers. To this end, COS cells were transfected with hS100A9 alone, treated with IL1β and subsequently with DSS. After Western blot analysis, we detected a 28 kDa band in the sample treated with IL1β but not in the untreated control, confirming that hS100A9 was rescued from protein degradation by IL1β, via formation of protease resistant homodimers (Fig. 7a, lane 2).

## Discussion

In the present work, we investigated the stability of the different oligomers formed by hS100A8 and hS100A9 in the myeloid cell line THP-1 and in transfected COS cells. We have shown that hS100A8 and hS100A9 could form homo- and heterodimers *in vivo*. While the hS100A8/hS100A9 heterodimer was the most stable oligomer, hS100A9 homodimers were unstable and barely detectable by Western blot. However, proteasome inhibition or inflammatory stimuli such as IL1β and TNFα could promote the formation of protease-resistant hS100A9 homodimers.

It is well accepted that the predominant form in which S100A8/S100A9 associates in physiological and pathological conditions is the heterodimer, even if it has been observed that S100A8 and S100A9 could also form homodimers [34], [35]. An increasing amount of data indicate that S100A8 as well as S100A9 homodimers are important regulators of inflammation and cancer, exhibiting strong pro-inflammatory activity in various mouse models of diseases [36]–[38]. In particular it has been shown that, S100A8 homodimers could promote chondrocyte-mediated cartilage destruction, upon metalloproteinase activation, in experimental arthritis [38]. In addition, CD8^+^ cells from subjects with lupus erythematosus stimulated with S100A8 or S100A9 showed an upregulation of IL-17 expression, leading to the development of auto-reactive lymphocytes [37]. Moreover, it has been shown that murine S100A8 homodimers were able to recruit leukocytes and had properties of an oxygen scavenger [39].

On the other hand, it has been observed that S100A9 homodimers interacted with TLR4 and RAGE [40], which are two receptors involved in the control of tumour growth in different systems [41], [42]. In particular, it has been shown that inhibition of hS100A9/TLR4 interaction inhibited tumour growth [29]. In addition, hS100A9 is involved also in metastasis formation, most likely by interfering at an early stage of metastasis formation [43]. We have shown that treatment with an S100A9-binding molecule inhibited metastasis formation in a prostate cancer tumour model [44]. It has been observed that S100A9 was important for the development and function of myeloid-derived suppressor cells (MDSC) [45], [46]. Also, our previous data has shown that human S100A9 was a TLR4-dependent pro-inflammatory molecule, activating NFκB in monocytes [47]. All together, these findings pointed out that S100A9, rather than S100A8/S100A9, could be the main mediator of inflammatory diseases and tumours and, more importantly, is emerging as a potential target for the treatment of malignant diseases.

Despite the emerging importance attributed to S100A8 and S100A9 homodimers, the most abundant form detected in serum is the heterodimer. Thus, in rheumatoid arthritis patients the amounts of heterodimer were 1000 fold greater as compared to S100A8 and S100A9 homodimers [21]. In addition, it has been shown with a two-hybrid system that hS100A8 and hS100A9 could not form homodimers in yeast [48]. Lastly, in granulocytes, the S100A8/S100A9 heterodimer was protease resistant while the S100A8 and S100A9 homodimers were not [31].

In our work, we showed by Western blot analyses that in THP-1 cells, no hS100A8 and hS100A9 homodimer could be found and we confirmed that the S100A8/S100A9 heterodimer was protease-resistant both in THP-1 and in COS cells over-expressing these proteins. However, we also showed that hS100A9 homodimers seemed to be rapidly degraded in COS cells, while hS100A8 homodimers were perfectly stable, which is in conflict with previously published data [31], [48]. This apparent discrepancy could be due to the presence of different subsets of proteases in COS cells, yeasts and extracellular milieu, leading to a different hS100A8 protein turnover.

We speculate that hS100A9 degradation occurred via ubiquitination and proteasomal degradation, since hS100A9 protein expression could be rescued by the proteasomal inhibitor MG132. Using UbPred program (predictor of protein ubiquitination sites) we found that Lysine 93 (K93) of the hS100A9 protein could be a target for ubiquitination.

We also showed that hS100A9 homodimers could be stabilized by forming protease-resistant homodimers in cells exposed to inflammatory stimuli. We confirmed our data also in a fibroblast cell line. That inflammatory conditions could promote hS100A9 homodimer formation, and that hS100A9 is in itself a pro-inflammatory signal, suggests a mechanism for an inflammatory amplification step where an inflammatory cytokine would trigger the production of an additional pro-inflammatory signal. Further studies will be needed to dissect the detailed mechanism by which inflammatory stimuli stabilized hS100A9 homodimers. One possible explanation for the formation of protease-resistant hS100A9 homodimers could be due to the fact that, upon inflammatory stimuli, hS100A9 could potentially be post-translationally modified. Indeed, it is well established that S100 proteins can be subject to several post-translational modifications [49].

In summary, in this work we showed that hS100A9 homodimer, which was rapidly proteolytically degraded, could be rescued by inflammatory stimuli and co-expression with hS100A8. The stabilization of hS100A9 homodimers may allow hS100A9 to interact with its target receptors TLR4 and/or RAGE that, in turn, could start an intracellular signal cascade, mediating hS100A9 homodimer effects, which could be distinct compared to effects induced by S100A8/S100A9 heterodimers.

## Supporting Information

Figure S1 **THP-1 and COS cells viability.** THP-1 (**A**) and COS (**B**) cells were treated with 10 and 100 ng/ml of the protein translation inhibitor cycloheximide respectively. Untreated and treated cells were collected at 4 h, 24 h and 48 h and subsequently counted using trypan blue dye.
(EPS)

Figure S2 **TNFα induced hS100A9 protein stabilization.** COS cells were transfected either with hS100A8 or hS100A9 constructs, as described above. 24 h after transfection, COS cells were stimulated with either 1 or 10 ng/ml TNFα. Then, samples were collected and analyzed by Western blot. Filters were stained with (**A**) anti-hS100A9 and (**B**) anti-hS100A8. Lane 1 (**NT**) represented non-transfected cells; from lane 2 to 4, COS cells were transfected with (**A**; **A9 T**) hS100A9 or (**B**; **A8 T**) hS100A8 separately; in lane 5 (**A8/A9 T**) COS cells were co-transfected with both plasmids.
(EPS)
